# The effect of individualized NUTritional counseling on muscle mass and treatment outcome in patients with metastatic COLOrectal cancer undergoing chemotherapy: a randomized controlled trial protocol

**DOI:** 10.1186/s12885-015-1092-5

**Published:** 2015-03-05

**Authors:** Anne van der Werf, Susanne Blauwhoff-Buskermolen, Jacqueline AE Langius, Johannes Berkhof, Henk MW Verheul, Marian AE de van der Schueren

**Affiliations:** 1 Department of Nutrition and Dietetics, Internal Medicine, VU University Medical Center, Amsterdam, The Netherlands; 2 Department of Medical Oncology, Internal Medicine, VU University Medical Center, Amsterdam, The Netherlands; 3 Faculty of Health, Nutrition and Sport, The Hague University of Applied Sciences, The Hague, The Netherlands; 4 Department of Epidemiology en Biostatistics, VU University, Amsterdam, The Netherlands; 5 Faculty of Health and Social Studies, Department of Nutrition, Sports and Health, HAN University of Applied Sciences, Nijmegen, The Netherlands

**Keywords:** Colorectal cancer, Malnutrition, Muscle mass, Nutritional counseling, Quality of life, Treatment toxicity, Survival

## Abstract

**Background:**

A low muscle mass is prevalent in patients with metastatic colorectal cancer (mCRC) and has been associated with poor treatment outcome. Chemotherapeutic treatment has an additional unfavorable effect on muscle mass. Sufficient protein intake and physical activity are known to induce muscle protein anabolism in healthy individuals, however it is unclear whether optimal nutrition is effective to preserve muscle mass in patients with mCRC during first-line chemotherapy as well. We hypothesize that individual nutritional counseling by a trained dietitian during first-line chemotherapy is effective in preserving muscle mass and may improve clinical outcomes in patients with mCRC.

**Methods/Design:**

In this multi-center single-blind randomized controlled trial, patients with mCRC scheduled for first-line combination chemotherapy consisting of oxaliplatin and fluoropyrimidine, with or without bevacizumab (n = 110), will be randomized to receive either individualized nutritional counseling by a trained dietitian to achieve a sufficient dietary intake and an adequate physical activity level, or usual care. Outcome measures will be assessed at baseline and after two and four months of treatment. The primary endpoint will be the change in skeletal muscle area (measured by CT-scan) at the first treatment evaluation. Secondary endpoints will be quality of life, physical functioning, treatment toxicity, treatment intensity and survival. Statistical analyses include one-sided t-tests for the primary endpoint and mixed models and the Kaplan-Meier method for secondary endpoints.

**Discussion:**

This randomized controlled trial will provide evidence whether individualized nutritional counseling during chemotherapy is effective in preventing loss of muscle mass in patients with mCRC.

**Trial registration:**

ClinicalTrials.gov NCT01998152; Netherlands Trial Register NTR4223.

## Background

Colorectal cancer is the third most common cancer in the world with nearly 1.4 million newly diagnosed patients in 2012 [1]. In 20% patients have metastatic disease at diagnosis and approximately 50% of the patients develops metastatic disease (stage IV colorectal cancer) [2]. For patients with disseminated disease for which local treatment with curative intent is not possible, the aim of treatment is to prolong survival with a good quality of life. Current combination treatment regimens of chemotherapy and targeted agents result in a in a median survival up to 23–31 months [3-5].

Malnutrition and weight loss are common problems in patients with metastatic colorectal cancer (mCRC) [6-8]: the prevalence of any self-reported weight loss at presentation varies from 34 to 72% [7,9] and 32% of the patients have lost more than 10% of their body weight at presentation [8]. In addition to the loss of total body weight, disproportionate loss of lean tissue weight is common in patients with cancer [10]. A previous study described a low muscle mass in 39% of the patients with mCRC [8]. In our own institution we observed a low muscle mass in 57% of the patients, while further loss of muscle mass during treatment was present in more than half of the patients (unpublished data), potentially due to a decreased nutritional intake as a consequence of chemotherapeutic toxicity [11]. In addition, physical activity has shown to be decreased during treatment [12,13], which could accelerate loss of muscle mass [14,15] and is related to muscular deconditioning [16].

The relevance of muscle mass in patients with cancer undergoing chemotherapy treatment has been described in several studies. Observational studies show that an unfavorable body composition with a low muscle mass is associated with reduced functional status [17] and quality of life [18], more severe toxicity of treatment [19,20] and reduced survival [8,15,17,21,22]. A potential explanation for a low muscle mass being an adverse prognostic factor is that a low muscle mass reflects the increased metabolic activity of a more aggressive tumor biology [22,23], although the underlying mechanism explaining this association has yet to be determined [22]. Another possibility is that patients with a low muscle mass are more fragile and susceptible to medical events [24], leading to a higher incidence of chemotherapy-related toxicity [25] and to suboptimal treatment (delay, reduction or interruption of chemotherapy) [24], both potential contributors to reduced survival [26]. In this case, clinical outcomes may be improved by interventions aiming at preserving muscle mass. For inducing muscle protein anabolism, a sufficient protein intake, next to an adequete physical activity, is of critical importance [14,27,28].

Only a few randomized controlled trials have been performed to evaluate nutritional interventions in patients with mCRC, none of them describing the effect of nutritional intervention on muscle mass. One study suggested that dietary advice had a beneficial effect on body weight after one year, although patients with different types of tumors were included and the numbers involved were small (n = 68) [29]. Another randomized controlled trial was ended prematurely because of crossover between the intervention- and control arm [7]. A third study showed a beneficial effect of parenteral nutriton compared to intensive enteral nutrition on body mass index (BMI), body cell mass, quality of life, chemotherapy-associated toxicity and survival (n = 82), but there was no comparison to placebo [30]. Due to absence of concrete evidence for a beneficial effect of nutritional intervention on muscle mass and treatment outcomes, there are no clear guidelines for nutritional support in this selected population. This is the main reason that additional nutritional support is not always provided [31].

We designed a randomized controlled trial to test our hypothesis that individual nutritional counseling (NC) by a trained dietitian during first-line chemotherapy is effective in preserving muscle mass and thereby may improve clinical outcome in patients with mCRC. The main objective of the study is to determine whether NC is effective in preserving muscle mass in patients with mCRC during chemotherapy. In addition, treatment toxicity, quality of life and survival will be evaluated.

## Methods

This single-blind multi-center randomized controlled study will be performed by the Departments of Nutrition and Dietetics and Medical Oncology of the VU University Medical Center Amsterdam, The Netherlands. Patients will be recruited from at least two Dutch hospitals (VU University Medical Center, Amsterdam and Spaarne Hospital, Hoofddorp), additional hospitals will be asked for participation. Ethics approval has been obtained from the Medical Ethical Committee of the VU University Medical Center. This study will be conducted according to the principles of the Declaration of Helsinki (64^th^ version, October 2013) and in accordance with the Medical Research Involving Human Subjects Act (WMO, 1-3-2006).

### Study population

Patients diagnosed with mCRC and scheduled for first-line chemotherapy with capecitabine and oxaliplatin (CAPOX) or infusional 5-fluorouracil and oxaliplatin (FOLFOX), with or without bevacizumab (−B), will be invited to enter the study. All patients should be over 18 years of age, have a World Health Organization (WHO) performance score of 0–2, understand the Dutch language and be able and willing to give written informed consent. Exclusion criteria are chemotherapy in the previous three months and long-term use of high dose of corticosteroids (at least 3 weeks a dose of ≥10 mg prednisolone or equivalent).

**Figure 1:**
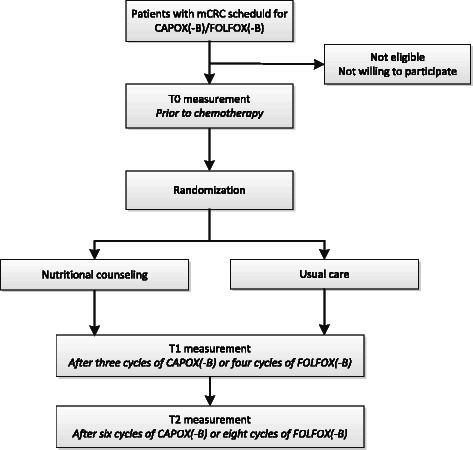
**Study flowchart.** mCRC: metastatic colorectal cancer.

Once enrolled, the patient will be randomized to receive either individual NC by a trained dietitian (n = 55) or usual nutritional care (UC, n = 55) during treatment with CAPOX(−B) or FOLFOX(−B) (Figure 1). If chemotherapy is stopped prematurely, study participation will also end. NC will be continued after study participation when preferred by the patient (intervention group).

### Randomization

Patients will be enrolled by a medical oncologist. The data manager will randomize patients either to the intervention group or the control group with use of randomization lists generated by the statistician. Patients are randomized in blocks of two and stratified for participating center, type of chemotherapy and WHO performance score (0/1 versus 2).

### Blinding

The research assistant, who conducts the study visits and performs the measurements, is blinded to the group assignment of the patients. Patients are requested to withhold their group assignment to the research assistant. Due to the nature of the intervention, the treating dietitian, coordinating researcher and participants cannot be blinded. Skeletal muscle area measurement and data analyses will be performed after blinding for treatment allocation.

### Intervention – nutritional counseling

Patients who have been assigned to the intervention group will receive individualized NC by a trained dietitian, starting at the first cycle of chemotherapy. The main goals of the nutritional intervention will be to enable every patient to achieve at least sufficient protein- and energy intake with attention for sufficient intake of micronutrients and an adequate physical activity level as described below.

NC is planned shortly before every treatment cycle, with telephone reviews in between the face-to-face sessions. Counseling consists of stimulating a sufficient protein- and energy intake, based on the current ESPEN (European society for clinical nutrition and metabolism) guidelines for protein and energy [32]. The criterion for sufficient protein intake is at least 1.2 grams per kg body weight [33]. In patients with a BMI of >30 kg/m^2^, protein requirements will be adjusted to a BMI of 27.5 kg/m^2^ [34]. Furthermore patients are advised to use at least 25 grams of proteins per meal. This evenly distributed ingestion of protein throughout the day is expected to maximally stimulate muscle protein synthesis [27]. Energy requirements are calculated based on the estimated resting energy expenditure of Harris and Benedict [35] plus an additional factor of 30% to correct for activity and disease.

To achieve a sufficient intake, an energy- and protein enriched diet using regular food will be advised and easy snack ideas and recipe suggestions will be provided. If a patient is unable to meet the dietary recommendations (less than 75% of energy- and/or protein needs) and/or loses body weight of ≥1 kg during a chemotherapy cycle, energy- and protein enriched oral nutritional supplements will be provided. If the body weight continues to decrease or if nutritional goals cannot be met in spite of oral nutritional supplements, tube feeding is indicated.

In addition to nutritional counseling, the dietitian will encourage patients to achieve a physical activity level according to the Dutch Healthy Exercise norm: at least half an hour of moderately intensive physical activity (e.g. walking, cycling or swimming) on at least five days per week.

### Control – usual nutritional care

Patients in the control-arm will receive UC: the medical oncologist observes on a regular base at the outpatient clinic and determines the patient’s tolerance, intake, condition and body weight as usual. When the medical oncologist concludes referral to a dietitian is indicated – for instance in case of severe weight loss or insufficient dietary intake – a dietitian will be consulted in agreement with the patient.

### Assessments

Outcomes will be assessed at study visits prior to chemotherapy (baseline, T0) and after three cycles of CAPOX(-B) (±9 weeks) or four cycles of FOLFOX(-B) (±8 weeks) (T1), when therapy response is evaluated by CT-scan. When chemotherapy is continued after T1, study outcomes will also be assessed after six cycles of CAPOX(-B) (±18 weeks) or eight cycles of FOLFOX(-B) (±16 weeks) (T2). If chemotherapy is stopped or switched to another chemotherapeutic drug after T1, T1 measures – among which the primary study endpoint – will be completed and study participation will be ended. Figure 1 shows a study flowchart and Table 1 gives an overview of all outcome measures.

**Table 1:** **Outcome measures**

Measurement	Instrument	Time point*
*Primary outcome*		
Skeletal muscle area	CT (skeletal muscle area L3)	T0, T1
*Secondary outcomes*		
Skeletal muscle area	CT (skeletal muscle area L3)	T0, T2
Quality of life	EORTC QLQ C30 (global health- and physical functioning domain)	T0, T1, T2
Hand grip strength	Hydraulic hand dynamometer	T0, T1, T2
Treatment toxicity	Common Toxicity Criteria version 4.0	ESP
Treatment intensity	Dose index and time index of chemotherapy	ESP
Adverse events	Medical record	ESP
Treatment response	Response Evaluation Criteria In Solid Tumors (RECIST)	T1, T2
Progression free and overall survival	Medical record or general practitioner office	After 2 years

*T0: prior to chemotherapy; T1: after three cycles of CAPOX(−B) or four cycles of FOLFOX(−B); T2: after six cycles of CAPOX(−B) or eight cycles of FOLFOX(−B); ESP: entire study period.

CT: Computed Tomography; EORTC QLQ: The European Organisation for Research and Treatment of Cancer: Quality of life questionnaire. L3: third lumbar vertebra.

#### Patient and treatment characteristics

Demographic variables like age, gender and living situation will be obtained from the medical record and a baseline questionnaire. Medical data include comorbidity (using the Charlson Comorbidity Index [36]), co-medication and WHO performance score and will be extracted from medical records.

#### Primary outcome

The primary endpoint will be the difference in change in skeletal muscle area during the first three cycles of CAPOX(−B) or four cycles of FOLFOX(−B) between the intervention- and the control group. Baseline computed tomography (CT)-scans (made within 30 days before start of chemotherapy) will be compared to CT-scans at T1 to determine change in skeletal muscle area, using routinely conducted CT-scans for diagnostic and disease evaluation purposes. A trained, blinded person will measure skeletal muscle area (cm^2^) with SliceOmatic software V5.0 (Tomovision, Canada). The image at the level of the third lumbar vertebra (L3) most clearly displaying both vertebral transverse processes will be chosen for measuring muscle area, since total cross sectional skeletal muscle area at this level is highly correlated with whole body skeletal muscle mass [37,38]. Slices of sequential CT-scans of one patient will be selected at the same time using a split screen to ensure a consistent location. Skeletal muscles at the level of L3 are identified based on anatomical features and quantified using Hounsfield units with thresholds for skeletal muscle tissue from −29 to +150 [39]. The sum of all these cross-sectional muscle areas (cm^2^) will be will be computed by summing tissue pixels and multiplying by the pixel surface area for each patient at each time point.

#### Secondary outcomes

Secondary outcomes of this study will be (change in) the following parameters between baseline (T0) and follow-up (T1, T2), comparing the NC-group with the UC-group:

##### Change in skeletal muscle area after completion of first-line chemotherapy

If chemotherapy is continued after three cycles of CAPOX(−B) or four cycles of FOLFOX(−B), change in skeletal muscle area at L3 will also be determined using CT-scans after six cycles of CAPOX(−B) or eight cycles of FOLFOX(−B). In addition, body composition will be estimated by bioelectrical impedance at each study visit to assess the association with change in muscle mass on CT-scan.

##### Quality of life

The European Organization for Research and Treatment of Cancer: Quality of life questionnaire (EORTC QLQ-C30) will be used to assess quality of life [40]. We have chosen to include the global health domain and the physical functioning domain as main items in our quality of life analyses. The other items will be analyzed in an explorative manner (including role-, emotional-, cognitive- and social functioning, the symptom scales, nausea and vomiting, pain, dyspnea, insomnia, loss of appetite, constipation, diarrhea and financial difficulties). Questionnaires will be scored according to the procedures specified by the EORTC [41].

##### Hand grip strength

Hand grip strength is an indicator of overall muscle strength and is associated with functional performance in advanced cancer patients [42,43]. Hand grip strength will be measured using a hydraulic hand dynamometer (Baseline, Fabrication Enterprises, USA) adjusted for the patient’s hand size. The test will be performed sitting, with the shoulder adducted and neutrally rotated, elbow flexed at 90 degrees, forearm and wrist in neutral position. The highest value of two maximal isometric contractions for each hand is recorded to the nearest kg. Measurements at different time points will be compared to estimate changes in muscle strength over time.

##### Treatment related outcomes

Treatment related outcomes include treatment toxicity, treatment intensity, treatment outcome and survival. During the entire study period, adverse events and treatment toxicity according to the Common Toxicity Criteria version 4.0 [44] will be monitored by the treating physician. Grade 3 to 5 toxicity when related to the treatment will be recorded as adverse side effects from treatment. Adverse events and serious adverse advents will be documented until study participation is ended. Treatment intensity will be subdivided in dose index (received cumulative dose/planned cumulative dose) and time index (planned duration of therapy/actual duration of therapy). Treatment outcome will be evaluated at T1 and T2 with use of the Response Evaluation Criteria In Solid Tumors (RECIST) [45] and is defined as complete response, partial response, stable disease and progressive disease. Furthermore, tumor marker carcinoembryonic antigen (CEA; μg/l) will be measured if initially elevated during routine blood sampling at least once every six weeks. Progression free survival and overall survival will be evaluated.

#### Other measures

##### Nutritional intake and physical activity

Nutritional intake and physical activity will be assessed concurrently during 3 days (one weekend- and two weekdays) at T0, T1 and T2 to evaluate compliance to the intervention.

Patients are asked to keep a 3-day food diary to reliably estimate nutritional intake [46]. During the study visit, this diary will be comprehensively checked on completeness by a trained and blinded research assistant. Daily dietary energy- and macronutrient intake and distribution of protein throughout the day will be calculated by a nutrition analysis software application with use of the most recent Dutch Food Composition table (NEVO, RIVM, Bilthoven).

Physical activity will be estimated using a calibrated physical activity monitor (PAM) accelerometer (model AM200, PAM B.V., Doorwerth, The Netherlands). The PAM scores physical activity based on acceleration and duration of the activity. Accumulation of all PAM-points during a day results in a PAM score, which indicates daily physical activity and is a valid measure for habitual physical activity [47].

##### Blood sampling

In addition to CEA, inflammation marker C-reactive protein will be measured during routine blood sampling at T0, T1 and T2. Furthermore, one sample of stored serum and one sample of stored plasma will be collected at T0 and T1 for future analysis on serum proteins.

### Sample size

Sample size calculations were made based on demonstrating a decline in the proportion of patients showing a clinically relevant decrease in skeletal muscle area of 6.0 cm^2^ (corresponding with approximately 1 kg loss of skeletal muscle mass) [38,48]. To achieve 80% power with a one-sided t-test for difference in proportions (α = 0.05), a sample size of 100 patients is required (assuming a standard deviation of 9.5 cm^2^ and a mean decrease in skeletal muscle area of 6.5 cm^2^ in the control arm and 0 cm^2^ in the intervention arm). A 10% buffer is added to account for loss to follow-up before the clinical endpoint can be assessed, resulting in a total sample size of 110 patients, 55 per study arm.

### Statistical analysis

Data will be analyzed using SPSS (IBM Corp. Armonk, NY) for descriptive- and statistical analyses. All analyses will be performed according to the intention-to-treat principle. For the primary outcome, one-sided t-tests for difference in proportions will be performed to compare the proportion of patients with a clinical relevant decrease in skeletal muscle area (6.0 cm^2^) between the NC- and the UC-group. Difference in change in skeletal muscle area will also be assessed performing independent t-tests. For secondary outcomes, mixed effect models will be used to evaluate change over time in dietary intake, physical activity, hand grip strength, quality of life and treatment related outcomes and to examine differences between groups. Furthermore the association between dietary intake/physical activity and skeletal muscle area will be assessed using regression models. Survival probabilities will be estimated with the Kaplan-Meier method.

## Discussion

Malnutrition is a prevalent and underrecognized problem in patients diagnosed with colorectal cancer. Of the patients with mCRC, 39-57% already has a low muscle mass at diagnosis [8] and these patients are at risk of further loss of muscle mass during chemotherapy. Observational studies show that a low muscle mass is associated with an adverse prognosis in patients with cancer. When poor outcome is a consequence of a low muscle mass – possibly by less treatment tolerance leading to suboptimal treatment intensity and reduced survival – interventions aiming at preserving muscle mass may improve clinical outcomes. To date no randomized controlled trial has been performed to study the effect of NC on muscle mass in patients with mCRC undergoing chemotherapy.

This study will determine the effect of NC (focused on a sufficient dietary intake and an adequate physical activity level) on muscle mass during first-line chemotherapy. The main objective is to evaluate whether NC can help to preserve muscle mass. As secondary outcome measures, this study will also evaluate whether preservation of muscle mass may improve the clinical outcomes such as quality of life, physical functioning, treatment toxicity and progression free survival.

The present study could provide an evidence based support for the potential effect of NC. If this randomized controlled trial demonstrates a beneficial effect of NC on its primary outcome muscle mass in patients with mCRC, NC should be evaluated in a subsequent phase 3 trial powered to determine whether it improves progression free and overall survival as well as quality of life.

## Abbreviations

BMI: Body mass index
CAPOX(−B): Combination chemotherapy of capecitabin and oxaliplatin (and – bevacizumab)
CEA: Carcinoembryonic antigen
CT: Computed tomography
EORTC QLQ-C30: The European Organisation for Research and Treatment of Cancer: Quality of life questionnaire
FOLFOX(−B): Combination chemotherapy of 5-fluorouracil, leucovorin and oxaliplatin (and –bevacizumab)
L3: Third lumbar vertebra
mCRC: Metastatic colorectal cancer
NC: Nutritional counseling
PAM: Physical activity monitor
UC: Usual nutritional care
WHO: World Health Organization
